# Risk of pre-term births and major birth defects resulting from paternal intake of COVID-19 medications prior to conception

**DOI:** 10.21203/rs.3.rs-59420/v1

**Published:** 2020-08-26

**Authors:** Silvia Rizzi, Maarten Jan Wensink, Rune Lindahl-Jacobsen, Lu Tian, Ying Lu, Michael Eisenberg

**Affiliations:** University of Southern Denmark; Syddansk Universitet; Syddansk Universitet; Stanford University School of Medicine; Stanford University School of Medicine; Stanford University School of Medicine

**Keywords:** COVID-19, paternal reproductive safety, pharmacological treatment, pre-term birth, congenital malformation

## Abstract

**Objective.:**

With the ongoing COVID-19 pandemic, large numbers of people will receive one of the several medications proposed to treat COVID-19, including patients of reproductive age. Given that some medications have shown adverse effects on sperm quality, there might be a transgenerational concern. We aim at examining the association between drugs proposed to treat COVID-19 when taken by the father around conception and any pre-term birth or major birth defects in offspring in a nation-wide cohort study using Danish registry data. Offspring whose father filled at least one prescription of the following medications in the three months preceding conception were considered exposed: chloroquine, hydroxychloroquine, losartan, azithromycin, naproxen, dexamethasone and prednisone.

**Results.:**

For azithromycin and naproxen, large numbers of offspring were exposed (> 1800 offspring), and we found no association with adverse birth outcomes. For chloroquine, losartan and dexamethasone, exposure was intermediate (~ 900 offspring), and there was no statistically significant association with birth defects. For hydroxychloroquine and prednisone, exposure was limited (< 300 offspring). There is strong evidence that azithromycin and naproxen are safe with respect to pre-term birth and birth defects. With some caution, the other drugs investigated can be considered safe.

## Introduction

To find an effective treatment for COVID-19 several existing drugs have been assessed and approved for clinical trials through accelerated procedures worldwide^1,2^. These drugs meant to treat non COVID-19 specific conditions are now given massively to patients affected by COVID-19. In particular, recent results from a study on dexamethasone seem encouraging^3^.

Evidence suggests that males are more severely affected by the ongoing pandemic irrespective of age^4^ and therefore are exposed to more proposed treatments. Importantly, while most COVID-19 patients who require treatment are relatively older, the scale of this pandemic suggests that patients also include reproductive aged men and women.

We aimed to investigate the safety of COVID-19 drugs taken by fathers prior to conception to assess for adverse birth outcomes. A few recent studies have shown adverse effects of drugs, including chloroquine, on gametes^5–7^, raising a potential transgenerational concern. In the present cross-sectional study we analyze the association between the use of COVID-19 medications used by fathers three months before conception and pre-term birth and congenital malformations, using the Danish National Birth, Patient and Prescription Registries^8–10^ from 1997 to 2017.

## Methods

### Population

The Danish Birth Registry (MFR)^8^ records information on all children born in Denmark including the father (if known), the mother, and basic vital information, such as Apgar score, birth weight, birth length, and gestational age. We restricted the analysis to live born singletons for the time frame 1997–2016 (1,201,131 births).

The Danish National Patient Registry (DNPR)^9^ provides administrative and clinical data of all patients discharged from Danish hospitals and outpatient clinics. One primary and optional secondary diagnoses are recorded according to the International Classification of Diseases for each visit. Using the CPR number of the Danish Birth Registry, data of the Danish National Patient Registry were linked at the patient level for all children. Allowing one year after birth for follow-up, we identified all newborns who were found to have at least one major birth defect as per EUROCAT Guide 1.4^11^.

The Danish Birth Registry can be linked to the Danish National Prescription Registry (LMDB)^10^ through the CPR number of the father. This registry includes all medications prescribed by a medical doctor. We examined the medications that are being trialed as treatments for COVID-19. The list of medications was retrieved from the Danish Medicines Agency^12^. We included those drugs with adequate usage to have a reasonable power: chloroquine, hydroxychloroquine, losartan, azithromycin, naproxen, dexamethasone and prednisone. We examined medications taken in the three months prior to conception based on a spermatogenesis cycle. Using the CPR number of the father, also the birth day, income, and the highest educational attainment of the father was retrieved from the population and education registries held at Statistics Denmark.

### Outcome

Our two primary outcomes were odds of pre-term birth (birth at less than 37 weeks of gestation) and of having at least one major birth defect in offspring (as per EUROCAT Guide 1.4^12^).

### Statistical analysis

All analyses were carried out at the secure server of Statistics Denmark in R (version 3.6.1)^13^. Conception date was calculated as the birth date minus gestation age as held in the registry. Infants born to fathers who had at least one prescription within three months before conception were considered exposed. Education of the father was simplified to three levels: low, middle and high. Smoking status of the mother during pregnancy was reduced to three levels: no smoker, past smoker, and current smoker. To handle missing data (< 5%) we created five imputed datasets using multiple imputation by chained equations with the R package *mice* (version 3.6.1) using predictive mean matching for the numeric variables and polytomous regression for the categorical variables. We performed logistic regression on each of the medications separately, adjusting for birth year, the age of both parents, smoking status of the mother, and education and income of the father. In addition, the regressions of major birth defects were adjusted for gestation age. We also performed logistic regression with all medications as independent variables simultaneously. The statistical significance threshold for all tests is set at two-side 0.05 level.

## Results

The average age of fathers was 32.3 years, while mothers were on average 29.7 years old. Of the mothers, 84.1% did not smoke at conception, 2.6% quit during pregnancy and 13.3% smoked during pregnancy. Of the fathers, 79.9% had at least upper secondary education, of which 11.8% had at least a masters degree.

Medications taken by fathers in significant amounts (> 100 fathers) were chloroquine, hydroxychloroquine, losartan, azithromycin, naproxen, dexamethasone, and prednisone.

The overall birth defect rate was 3.2%, while 7.4% of all births were preterm.

None of the investigated drugs taken by men up to three months before conception was associated with pre-term birth or congenital malformations (Table 1). Including all the medications in the same regression model yielded identical results.

For hydroxychloroquine and prednisone, exposure was limited (< 300 offspring). For azithromycin and naproxen, large numbers of offspring were exposed (> 1800), and we found no association with adverse birth outcomes. For chloroquine, losartan and dexamethasone, exposure was intermediate (~ 900 offspring), while there was a no statistically significant association with birth defects.

## Discussion And Conclusion

Using a nationwide cross-sectional study with data over 20 years in Denmark, we found no evidence of association between the COVID-19 drugs under study, taken by men up to three months before conception, and pre-term birth or major birth defects in offspring. While reassuring, given the limitations of the current report, continued surveillance of parental exposure to experimental COVID-19 treatments remains prudent.

## Limitations

The prescription registry does not include prescription diagnosis. It was therefore not possible to separate the effect of the medication from the effect of the disease for which the medication was given. This study will therefore tend to overestimate, but not underestimate any effect. This might (partly) explain odds ratios larger than one for chloroquine and losartan. In addition, medications that can be obtained over the counter (e.g. naproxen) may be misclassified biasing results toward the null.

## Figures and Tables

**Table 1:** Paternal medication taken around conception in Denmark 1997–2017 and births (singleton and alive), full term, pre-term, and with birth defects: Summary of data and results of logistic regression model.

Medication	ATC Code	Births	Full Term	Pre-Term (%)	Birth Defect (%)	Pre-term vs Full Term		Birth Defect vs Not	
		1.201.131	1.087.841	86886 (7.4%)	38881 (3.2%)				
						OR (95% CI)	p-value	OR (95% CI)	p-value
Azithromycin	J01FA10	7278	6721	557 (7.7%)	235 (3.2%)	1.01 (0.93–1.10)	0.77	0.98 (0.86–1.12)	0.76
Naproxen	M01AE02	1895	1749	146 (7.7%)	63 (3.3%)	1.01 (0.85–1.20)	0.9	1.05 (0.82–1.35)	0.69
Losartan	C09CA01	972	865	69 (7.4%)	43 (4.6%)	1.01 (0.79–1.29)	0.96	1.30 (0.96–1.77)	0.094
Dexamethasone	H02AB02 S01BA01 S03BA01	907	838	69 (7.6%)	32 (3.5%)	1.05 (0.82–1.34)	0.72	1.11 (0.78–1.58)	0.57
Chloroquine	P01BA01	849	789	60 (7.1%)	30 (3.5%)	0.98 (0.75–1.28)	0.89	1.32 (0.91–1.90)	0.14
Prednisone	H02AB07	289	262	27 (9.3%)	9 (3.1%)	1.26 (0.85–1.87)	0.25	1.00 (0.51–1.94)	0.99
Hydroxychloroquine	P01BA02	101	> 95^#^	< 6^#^	< 6^#^	0.38 (0.12–1.21)	0.1	1.22 (0.45–3.32)	0.7

Exposure: Male preconception three months.

Births, Full Term, Pre-Term, and Birth Defect columns contain counts and percentage in brackets.

Logistic model adjusted for age of mother, age of father, birth year, education and income of the father, smoking status of the mother. Birth defects additionally adjusted for gestation age.

# Privacy regulations of Statistics Denmark do not allow publication of numbers smaller than 6.

## Abbreviations

MFR: Danish Birth Registry
DNPR: Danish National Patient Registry
LMDB: Danish National Prescription Registry
CPR: Central Person Register
EUROCAT: European Registry of Congenital Anomalies and Twins
